# Measuring Integration Processes in Visual Symmetry with Frequency-Tagged EEG

**DOI:** 10.1038/s41598-018-24513-w

**Published:** 2018-05-03

**Authors:** Nihan Alp, Peter Jes Kohler, Naoki Kogo, Johan Wagemans, Anthony Matthew Norcia

**Affiliations:** 1 Brain & Cognition, KU Leuven, Leuven, Belgium; 2 Department of Psychology, Stanford University, Stanford, USA; 3 Biophysics, Donders Institute for Brain, Cognition and Behaviour, Radboud University, Nijmegen, The Netherlands

## Abstract

Symmetry is a highly salient feature of the natural world which requires integration of visual features over space. The aim of the current work is to isolate dynamic neural correlates of symmetry-specific integration processes. We measured steady-state visual evoked potentials (SSVEP) as participants viewed symmetric patterns comprised of distinct spatial regions presented at two different frequencies (f_1_ and f_2_). We measured intermodulation components, shown to reflect non-linear processing at the neural level, indicating integration of spatially separated parts of the pattern. We generated a wallpaper pattern containing two reflection symmetry axes by tiling the plane with a two-fold reflection symmetric unit-pattern and split each unit-pattern diagonally into separate parts which could be presented at different frequencies. We compared SSVEPs measured for wallpapers and control patterns for which both images were equal in terms of translation and rotation symmetry but reflection symmetry could only emerge for the wallpaper pattern through integration of the image-pairs. We found that low-frequency intermodulation components differed between the wallpaper and control stimuli, indicating the presence of integration mechanisms specific to reflection symmetry. These results showed that spatial integration specific to symmetry perception can be isolated through a combination of stimulus design and the frequency tagging approach.

## Introduction

Symmetry is a fundamental principle of perceptual organization: symmetric configurations influence perceptual grouping, figure-ground segregation and object recognition^1–4^. Symmetry perception has been studied extensively behaviorally (for reviews, see refs^5–8^). Classic findings indicate that reflection symmetry is easier to detect compared to rotational or translational symmetry^9^ and that symmetric image features tend to be perceived as figures rather than as background^10^.

Although the characteristics of symmetry perception and its importance to perceptual organization are well-established at a functional level, little is known about its neural basis. Human electrophysiological and neuroimaging studies have provided information about when and where symmetry perception occurs in the brain. The first study of the neural basis of symmetry perception used electroencephalography (EEG) and showed that stimuli with reflection symmetry generated event-related potentials (ERPs) over occipital cortex with a sustained, negative polarity differential response at around ~220 ms after presentation of the stimuli, compared to random control stimuli^11^. This finding has been replicated and extended by many other studies^12–17^ and these negative responses to symmetry have been labelled as the sustained posterior negativity^14^ (SPN).

Two functional MRI studies using similar stimuli followed the initial EEG experiment. The first study^18^ reported strong responses to reflection symmetry in dorsal lateral occipital cortex (LOC). The second study^19^ extended these findings by showing that several extra-striate areas in visual cortex (LOC, ventral V4, V3A, V7) were sensitive to reflection symmetry. The perception of reflection symmetry was disrupted when the LOC was targeted by transcranial magnetic stimulation (TMS), showing that LOC plays a causal role in symmetry perception^20,21^. However, the primacy of LOC in perceiving symmetry has been challenged by a more recent study, which used a combination of fMRI and EEG that revealed rich representations of rotational symmetry are also present in retinotopically organized areas V3, hV4 and ventral occipital (VO1)^22^.

Functional MRI studies have thus localized symmetry processing to a large array of visual brain areas spanning all the way from the early visual area V3 (at least in the case of rotation) to higher-level visual area like LOC, while EEG studies have characterized the time course of the processes taking places in those areas. Despite these advances, neither approach have identified the spatial integration processes necessary for symmetry detection^23,24^. Here we expand on prior work by using a frequency-tagging approach that allows us to disassociate neural responses to the global configurations from responses to local features and, hence, to isolate the dynamic signatures of the integration processes that may take place during symmetry perception.

We adapted the multi-input frequency-tagging technique^25–27^ so that it could be used with patterns that contained controlled amounts and kinds of symmetry. To detect symmetry-related integration processes via the frequency-tagging approach, we split symmetric patterns into two diagonal parts and temporally modulated the parts at distinct temporal frequencies (f_1_ and f_2_). Visual stimulation at a specific temporal frequency generates steady-state visual evoked potentials (SSVEP) at the input frequencies (f_1_, f_2_) and at multiples (nf_1_ and mf_2_) of these frequencies. The responses that occur at multiples of the input frequencies (nf_1_ and mf_2_) are called harmonics or *self-terms*, because they reflect non-linear processes associated with each stimulus part. By applying two different frequencies to the separate parts of the image, we can capture non-linear interactions specific to spatial integration processes^28^. These interactions manifest at emergent frequencies that are not in the input, but are combinations of multiples of the input frequencies (f_1_ + f_2_, f_1_ − f_2_ etc.). These “intermodulation” or “IM” frequencies (nf_1_ ± mf_2_) are also called *mutual terms* because they reflect between-part non-linear interactions^29,30^.

IM components reflect integrative processes because they can only be generated by neural populations that non-linearly combine the stimuli presented at the two input frequencies. Investigations of the neural basis of integration processes in various perceptual phenomena have established IM components as an objective neural signature of integration processes occurring throughout the visual processing hierarchy^28,30–38^.

To study integrative processes underlying symmetry perception, we used symmetric patterns derived from the 17 wallpaper groups, a mathematically defined class of regular patterns that result from distinct combinations of the four fundamental types of symmetry operators: reflection, translation, rotation, and glide^39,40^. We selected one of the wallpaper groups, PMM, for use in our experiment for its particular combination of reflection and rotation symmetries. Another group, P2, contains only rotation symmetry and was used as a control condition. We also used a second control condition, which consisted of patterns that had no symmetries and were not wallpaper groups, but was feature-matched to the PMM and P2 patterns. The goal of the experiment was to isolate integrative processes specific to reflection symmetry via the intermodulation components of the two-input, frequency-tagged SSVEP.

**Figure 1:**
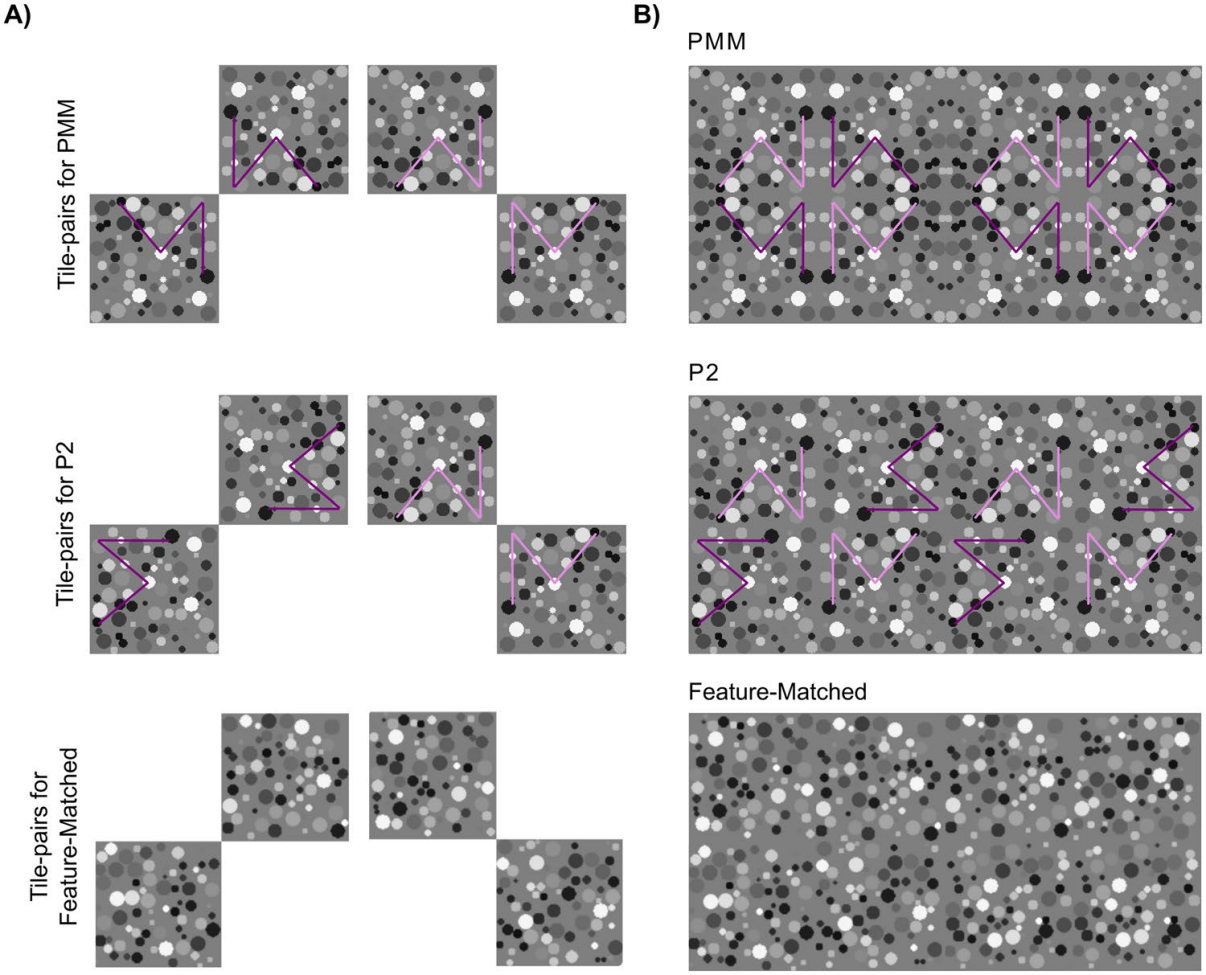
Tiles of all the patterns and three of the wallpaper pattern used in the experiment. (**A**) Tiles of each pattern type are shown. The local properties of individual dots (i.e. size, gray scale) were kept the same within the tile as well as across the three pattern types. (**B**) PMM, P2 and feature-matched patterns are shown. Note that both PMM and P2 included two 180° rotational symmetries within the diagonal parts of the unit-pattern, which were tagged with the same frequency. This is illustrated by the purple lines that can be seen within the diagonal pairs of both P2 and PMM tiles. The dark and light purple were used to differentiate the two different frequencies applied to diagonal halves and to highlight the rotation of one of the PMM tile pairs (dark purple) that is necessary for generating the P2 tiles. Please note that light and dark purple lines were not a part of the stimulus presentation, and are shown here to differentiate the two different frequencies applied to diagonal halves and to highlight the rotation of one of the PMM tile pairs (dark purple) that is necessary for generating the P2 tiles.

The PMM is a wallpaper group defined by 2 perpendicular reflection axes and four 180° rotations centered at the intersections of the reflection axes. We split the PMM pattern into two diagonal halves to tag with two different frequencies. Critically, each of these split halves independently contain only 180° rotations, but reflection symmetry emerges when the halves are combined into a complete pattern. To generate a PMM pattern, a “tile” (a quarter of the unit-pattern) was generated from a non-overlapping random dot pattern and a “unit-pattern” was constructed by arranging four identical (but rotated and/or flipped) tiles so that their combination gives rise to 2 perpendicular (vertical and horizontal) reflection axes (see Fig. 1).

The first control condition, P2, is a wallpaper group defined by 180° rotation symmetry, but no reflection. We generated a P2 for our control condition by first rotating the tile from the PMM pattern by 90° counterclockwise (compare Fig. 1A 1^st^ and 2^nd^ row), and then combining two diagonally split image-pairs, one from the original and one from the rotated PMM pattern. The result was a P2 pattern that preserved the 180° rotation within each diagonally split image pair from the original PMM, but had no reflection symmetry (Fig. 1B, 2^nd^ row). This means that the only difference between PMM and P2 is that PMM wallpaper pattern has emergent reflection symmetry.

Finally, we generated a feature-matched control by shuffling the position of each dot within a tile. The position was shuffled independently for each tile, across all unit-patterns. This preserves the local features (dots) from the original PMM pattern, but eliminates both global and local regularities, including all symmetries.

We applied separate frequency tags to the sub-regions of the PMM and control patterns in order to derive a differential measure of integration processes that take place during reflection symmetry perception. We used two frequency pairs in which the higher frequency was kept the same while the lower frequency was slightly lower for one pair than the other (Freq. Pair1 = 6 & 5 Hz; Freq. Pair2 = 6 & 4.29 Hz). This allowed us to test if the integration processes we measure were robust to slight changes in the input frequencies. We found that IM components over occipital cortex were enhanced in the PMM condition compared to the two control patterns, regardless of frequency pair. These findings have implications for understanding the neural dynamics of spatial integration during symmetry perception.

## Results

### Behavioral discrimination

Participants were instructed to report a pattern change from the base exemplar to another exemplar from the same pattern type by pressing a button on a joystick. Because of technical problems, we were unable to collect behavioral data for the pattern discrimination task from two participants, so the data reported here are from the 18 other participants. We computed percent correct and d’ for all participants and conditions. The percent correct was 85.38% (sd: 11.28) for discriminating different PMM wallpaper patterns (with reflection and rotation), 82.63% (sd: 12.63) for the two P2 wallpapers (with rotation only), and 78.71% (sd: 15.66) for the feature-matched control patterns.

**Figure 2:**
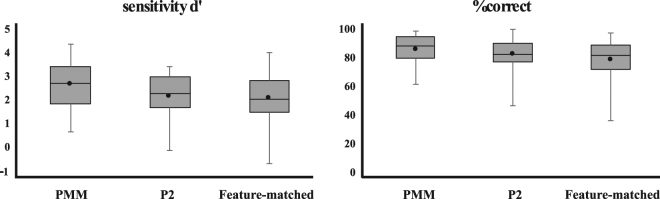
Behavioral performance. Percent correct for discriminating pattern exemplars and average d′ across 18 participants are shown. Black lines indicate the median while black dots indicate the mean. Error bars show variability outside the quartiles.

Each participant’s overall performance was above chance level d′ > 0 (Fig. 2). Only one participant showed below chance level performance for the P2 and feature-matched conditions d′ < 0. The average d′ was 2.28 (sd: 0.19) for PMM, 1.99 (sd: 0.17) for P2, and 1.68 (sd: 0.19) for PMM.

### Reliable Component Analysis – Frequency Domain

The reliable component analysis (RCA; for further details, see^41^) is a dimension reduction technique designed to provide components from a linear combination of electrodes which maximizes trial-to-trial spectral covariance. In SSVEP, the response phase is constant over repeated presentations of the same stimulus^26^. The RCA uses this across-trial reliability to decompose generalized eigenvalue problem into a small number of physiologically reliable components (RCs). Both for time and frequency domain, the RCA was computed separately for each frequency pair, and for the self and mutual terms, using data from all conditions (3 conditions per frequency pair).

We will first consider the results in the frequency domain. For the self-terms, the first two components explained a substantial amount of the trial-to-trial reliability for both frequency pairs (53% and 50%), and this was also true for the mutual terms (62% for both pairs). We restricted our analysis to the first two RCs, because that was the number of components required to explain the majority of the reliability in the mutual term data (~60%), and because we hypothesized that several distinct neural sources might be involved and wanted to be able to measure at least two of them, while at the same time not doing too many comparisons. If our approach can capture symmetry-specific spatial integration, we would expect to see differences in amplitude between the three pattern types (PMM, P2 and feature-matched) in the mutual terms for one or more of the RCs. We tested this by running two repeated-measures ANOVA on the RC amplitudes: one with pattern types (PMM, P2, feature-matched) and self-terms (f_1_, f_2_, 2f_1_, 2f_2_, 3f_1_, 3f_2_) as factors, and another with pattern types and mutual-terms (f_1_ − f_2_, f_1_ + f_2_, f_1_ − 2f_2_, 2f_1_ − f_2_, f_1_ + 2f_2_, 2f_1_ + f_2_, f_1_ + 3f_2_, 3f_1_ + f_2_, 3f_1_ − f_2_, 3f_2_ − f_1_). We did this separately for each frequency pair.

**Figure 3:**
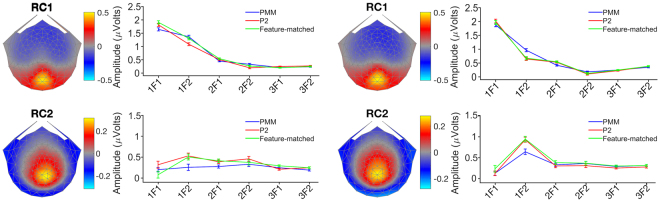
The RC topographies of self-terms and amplitude of two frequency pairs. The left column represents the first frequency pair, while the right column represents the second frequency pair. Rows represent two RCs. The first row shows topographies and amplitude values of RC1 from both frequency pairs. The second row shows topographies and amplitude values of RC2 from both frequency pairs. Error bars indicate the standard error of the mean.

The group-average amplitudes for the self-terms and the corresponding scalp topographies for each temporal frequency pair and RC are shown in Fig. 3. For the first RC, SSVEP amplitudes were maximal at the occipital pole and decreased monotonically with response frequency (order of harmonics) for both frequency pairs. The second RC had a dorsal-medial scalp distribution and had less amplitude variation across harmonics.

We used vector-based approach appropriate for complex-valued responses and computed the average phase and amplitude at stimulus frequency by averaging the real and imaginary part of the response separately across participants. We then calculated the vector mean amplitude and phase by combining the real and imaginary parts. We calculated a two-dimensional error ellipse by defining lower and upper bounds on the mean response amplitude. We calculated the longest and shortest vectors from the origin to the error ellipse by using a geometrical approach. This approach, which is explained in detail in^42^, captured the full range of response amplitudes within one standard error of the mean.

In order to determine the extent to which the signal at each of our components exceeded the noise floor, we used Hotelling’s *t*^2^ tests of the null hypothesis that the two-dimensional data set containing the real and imaginary parts of the complex value at the stimulus frequency was equal to [0, 0]^43^, on self and mutual terms for both frequency pairs (see Table 1). In this vector-based approach, both amplitude and phase, and their consistency across participants, contributes to our reported statistical significance.

**Table 1:** Significant and Insignificant Frequencies.

	Freq. Pair1				Freq. Pair2			
	RC1		RC2		RC1		RC2	
	Self Terms	Mutual Terms	Self Terms	Mutual Terms	Self Terms	Mutual Terms	Self Terms	Mutual Terms
PMM	***	***	***	***	***	**	***	**
				except		except	except	except
				1F1 + 3F2		3F1 + 1F2	1F1	−1F1 +3F2
				&				1F1 + 2F2
				3F1 + 1F2				1F1 + 3F2 & 3F1 + 1F2
P2	***	***	***	***	***	**	***	**
		except		except		except	except	except
		3F1-1F2		1F1 + 3F2		1F1 + 3F2	1F1	1F1-2F2
		&		&				1F1 + 2F2
		1F1 + 3F2		3F1 + 1F2				1F1 + 3F2 & 3F1 + 1F2
Feature-Matched	***	***	***	***	***	**	**	**
			except	except				except
			1F1	2F1 + 1F2				−1F1 + 3F2
				&				&
				1F1 + 3F2				1F1 + 3F2

***(p < 0.001) and **(p < 0.05) indicate that for all frequencies the Hotelling’s *t*^2^ test showed that the real and imaginary components of the response were significantly different from [0, 0]. The frequencies indicated in the tables are the ones which were not significant.

For self-terms, there were no main effects of pattern type for any combination of RC and frequency pair (Freq. Pair1 (RC1; F(2, 38) = 1.38 p = 0.26, RC2; F(2, 38) = 0.75 p = 0.47); Freq. Pair2 (RC1: *F*(2, 38) = 0.65, *p* = 0.53; RC2: *F*(2, 38) = 0.37, *p* = 0.69)). The interaction (pattern type × self-terms) was significant only for the combination of RC1 and Freq. Pair1 (*F*(4.41, 83.91) = 4.24, *p* = 0.003). Moreover, the topographies of RC1 and RC2 were similar across two different frequency pairs (Fig. 3).

**Figure 4:**
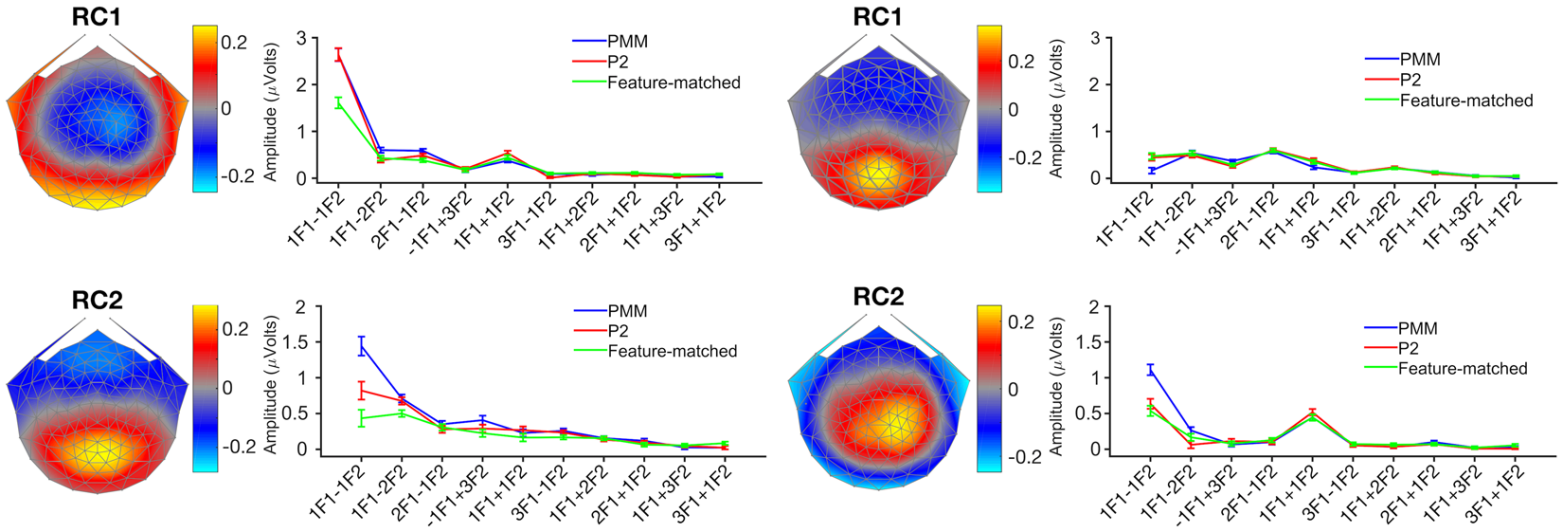
The RC topographies and amplitude of mutual-terms from two frequency pairs. The left column represents the first frequency pair, while the right column represents the second frequency pair. Rows represent two RCs. The first row shows topographies and amplitude values of RC1 from both frequency pairs. The second row shows topographies and amplitude values of RC2 from both frequency pairs. Error bars indicate the standard error of the mean.

The data for the IM components are shown in a similar format in Fig. 4. The topography of the first RC was distributed medially along the edge of the electrode array for the first frequency pair, but its maximum was displaced anteriorly for the second frequency pair. For the second RC, the scalp topographies were displaced anteriorly relative to their maxima for the first RC. The scalp distributions for the IM terms thus differed substantially between the two frequencies pairs and for both RCs, but those of the self-terms were similar within an RC for a given frequency pair. This suggested that the locations of the sources generating the non-linear interactions were more dependent on temporal frequency than those generating the self-terms.

We found significant differences between conditions only at IM frequencies. In three of the four comparisons, there were main effects of pattern type. For Freq. Pair1, we found a main effect of pattern type for the mutual terms for both RCs (RC1: *F*(2, 38) = 16.8, *p* < 0.001; RC2: *F*(2, 38) = 10.42, *p* < 0.001). Only RC2 showed a main effect of pattern type for Freq. Pair2 (*F*(1.52, 28.99) = 5.45, *p* = 0.015). The largest amplitude IM component was the difference frequency component (1F1-1F2) and it also showed the largest differential effects of pattern type. The pattern of these differences, however was complex and depended on the RC and the frequency pair. RC2 had the clearest differentiation of PMM from the other two conditions and this differentiation was present for both frequency pairs. Importantly, we found a significant interaction between pattern type and IM component in two of the four comparisons, indicating that the IM terms were sensitive to the type or presence of symmetry in the images. The interaction (pattern type × mutual-terms) effect was significant for RC1 for Freq. Pair1 (*F*(2.86, 54.42) = 11.96, *p* < 0.001), and for RC2 for Freq. Pair2 (*F*(4.12, 78.41) = 5.45, *p* < 0.001). Based on these findings, we concluded that the PMM and P2 conditions generated a differential response in most of the comparisons with the pattern that does not consist of any symmetry (feature-matched). Therefore, both frequency pairs elicited measurable responses that reflect spatial integration processes driven specifically by symmetry. Moreover, PMM also generated a differential response when compared to both P2 and feature matched condition in RC2 for both frequency pairs. This suggests that both frequency pairs also elicited measurable reflection symmetry specific responses.

### Reliable Component Analysis – Time Domain

The analysis described above indicated that some of the IM terms, but none of the self-terms, reflect the pattern type. However, analysis of frequency components one at a time does not reflect the full pattern of nonlinear interactions and does not take the phase of the components into account. Moreover, overall differences between conditions that are driven by combinations of several IM frequencies are difficult to detect and report in this way. Therefore, we converted the IM data from the frequency domain to the time domain using the inverse Fourier transform so that we could observe how the coherent summation of IM components differed between pattern types. We focused our analysis on the first two reliable components (RCs); their topographies were quite different across components, but similar for the two frequency pairs. To focus the analysis on differential responses to the different types, we calculated all pair-wise waveform differences between conditions. Considering the degrees of regularities in each pattern type, PMM (with reflection and rotation) >P2 (with rotation only) >feature-matched (with neither reflection nor rotation), it should be noted that the comparison between PMM and P2 is the only one which measures the integration processes specific to reflection symmetry. The comparison between PMM vs feature-matched could also indicate the integration processes of reflection symmetry, but this might be driven partially by the rotation symmetries present in the two PMM images, but not feature-matched. On the other hand, P2 vs feature-matched is likely to be driven by an increase in integration related to the rotation symmetries present in the two P2 images. This difference should be more pronounced for the maximally different patterns (PMM vs. feature-matched) and might be least pronounced for the minimal difference (P2 vs. feature-matched). We thus computed waveform differences and used permutation-based t-tests for the amplitude differences as a function of time, separately for (PMM − feature-matched; PMM − P2; P2 − feature-matched) and each frequency pair.

**Figure 5:**
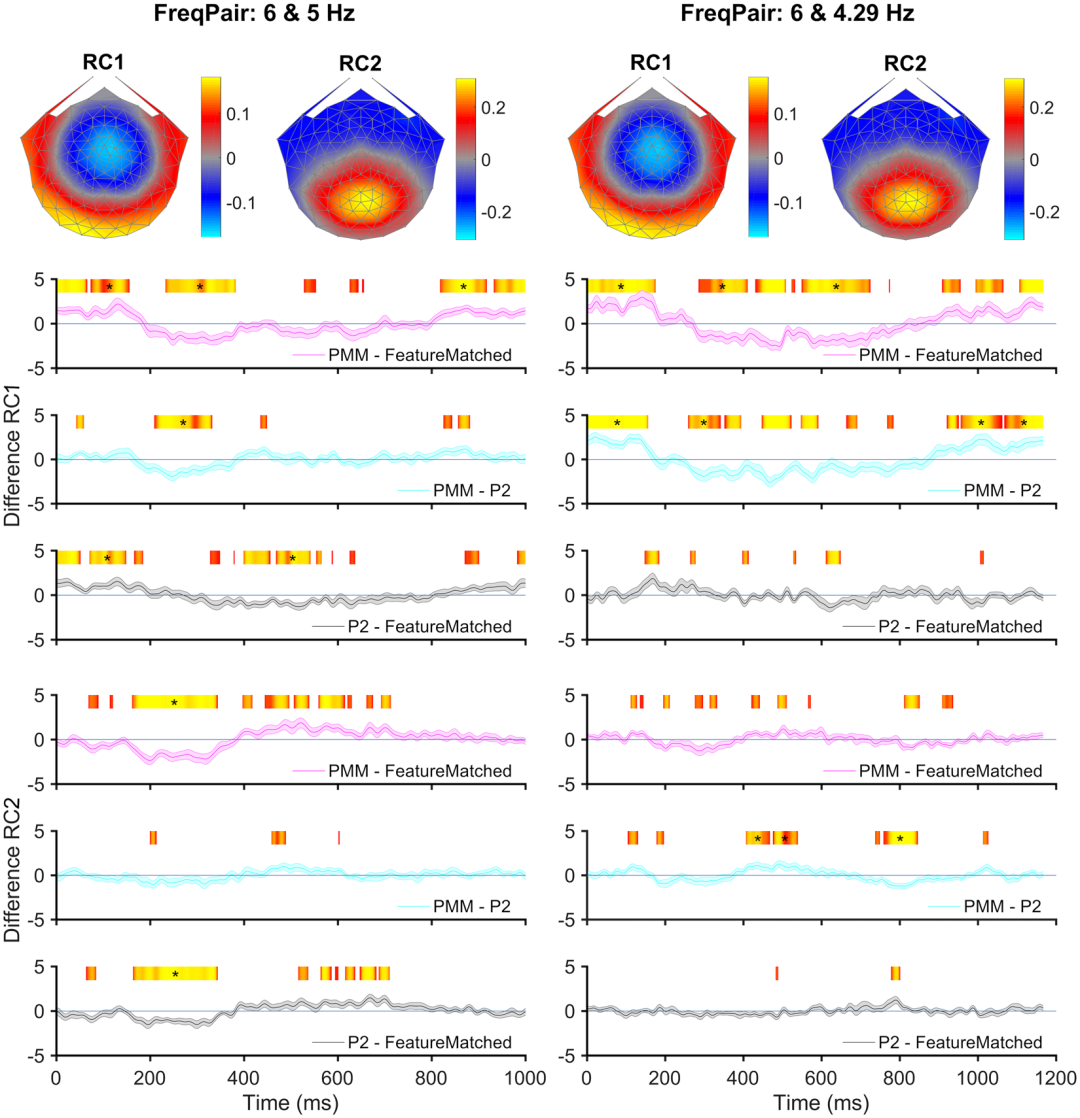
The time domain responses. Topographies and amplitude differences of two RCs for both frequency pairs are shown. The X axis represents the time for the mutual cycle of both frequencies per frequency pair. The Y axis shows the amplitude differences of RCs. Columns indicate two different frequency pairs, while rows indicate two RCs. The heat map is used to show the longest consecutive run of significant p values for a two-tailed t-test on difference RC amplitude separately. Difference RC amplitude was shown with pink (for PMM − feature-matched), cyan (for PMM − P2) and gray (for P2 − feature-matched) with error bars indicating the standard error of the mean. The heat map bars with a yellow-orange arrangement on top of each plot indicate uncorrected significant (p < 0.05) time points. Orange indicates p-values < 0.05, while yellow indicates smaller p-values. Asterisks are indicative of time points that are significantly longer than an expected chance level computed by the permutation test.

The first RC from both frequency pairs and, as well as the second RC from the first frequency pair, revealed a differential response for the predicted maximal difference (PMM − feature-matched). Moreover, differences between PMM and P2 were also significant in three of the four comparisons (see Fig. 5, 2^nd^ column: RC1 from both frequency pairs as well as RC2 from Freq. Pair2). We did not find significant differences between pattern types for the predicted minimal difference (P2 − feature-matched) in Freq. Pair2. Yet, this difference was significant in Freq. Pair1 for both RCs (see Fig. 5, 1^st^ column). Based on these findings, we concluded that the PMM condition generated a differential response when it was compared to either the feature-matched or the P2 controls in both frequency pairs. Therefore, both frequency pairs elicited measurable responses that reflect spatial integration processes driven specifically by reflection symmetry.

## Discussion

By using a direct measure of non-linear spatial interaction based on the IM components of the SSVEP and carefully controlled stimuli, we were able to isolate neural activity that reflects the global processing of reflection symmetry. With our experimental design, IM components can occur only as a result of non-linear integration of the two diagonal image pairs. In all conditions, the dots in the image parts that were tagged with two different frequencies were identical in terms of gray-scale content and size. For the PMM patterns, the combination of the image parts generated emergent reflection symmetries that were not present in each image-part individually. This was not the case for the P2 patterns; each image-part contains rotation symmetry of order two (180°), but the combination of the two parts did not give rise to any further rotation, reflection or any other symmetry. Finally, the feature-matched pattern had no symmetries in the individual image parts or in their combination. The specific comparison between the PMM and P2 conditions allowed us to disentangle non-linear interactions specific to the global processing of reflection symmetry, from non-linear interactions that might occur during perception of a highly regular pattern without reflection.

We thus expected stronger IM responses in the PMM condition, compared to the other two conditions, consistent with additional non-linear spatial interactions elicited by the presence of reflection symmetry. We did in fact see such a pattern in our measurements, as demonstrated by both the frequency and time domain analyses. We saw no differences between the self-terms for the three conditions, indicating our design was successful in matching the local features across conditions.

Visual inspection of the topographical distribution of RCs suggests that the topographies of the mutual terms are different from those of self-terms. The topographies of the self-term responses, especially RC1 for both frequency pairs (see Fig. 3), appear to be consistent with local processing in early visual cortex. In contrast, mutual-term responses, especially RC2 for both frequency pairs (see Fig. 4), have topographies that suggest sources outside of early visual cortex. The fact that significant IM response differences between PMM and P2 are primarily found in RC2 is consistent with the interpretation that RC2 captures processing outside of early visual cortex. IM response differences between PMM and P2 reflect global integration processes that are specific to reflection symmetry, and the neuroimaging literature indicates that processing of reflection symmetry mainly takes place outside early visual cortex.

In prior fMRI work, symmetry-related responses were found in LOC regardless of whether the stimulus consisted of symmetrical dot patterns^18,19^ or a symmetrical face^44^. The LOC also plays a role in tactile symmetry perception in the early blind^45^, and symmetry perception can be disrupted when TMS is applied to LOC (for a review see^46^). Several areas besides LOC have been identified as playing a role in symmetry processing^19,22,47^, but early visual areas V1 and V2 are likely not among them, with area V3 being the first area in the visual processing stream to show a consistent symmetry response^19^. This may be because only higher-tier visual areas have the large receptive fields required for the long-range spatial integration necessary for perceiving symmetry^48^. We did, however, see some evidence of an early visual cortex contribution to integration processes underlying symmetry perception: For Freq. Pair1 in RC1, PMM and P2 elicited differential responses when compared to the feature-matched condition, and this component had a topography that suggested signal generated in the early visual cortex, perhaps at the level of V3.

The magnitude of the IM terms decreases as a function of output frequency and is not specific to the non-linear order. This is demonstrated clearly for the two second-order response terms (1f_1_ ± 1f_2_). The difference frequency responses occurred at 1.0 and 1.71 Hz while the sum responses occurred at 11 and 10.29 Hz. In terms of non-linear order, these two terms are both 2^nd^ order with respect to the inputs. Despite the common non-linear order, the response amplitudes were very different. Thus, non-linear order is not simply predictive of responsiveness. Multi-frequency SSVEP IM responses have been interpreted in the context of simple cascade models comprised of a non-linearity placed between a linear filter at the input and a second linear filter at the output^49^. Within the context of this model, non-linearities such as squaring, rectification or normalization^49,50^ would be expected to produce both sum and difference frequency activity. The loss of the high-frequency sum term in this model, could be explained by a temporally integrating (low-pass) second filter that would eliminate the sum term generated by the prior nonlinearity in the cascade.

Perceiving symmetry not only requires a comparison across retinal locations but also requires integration of pairs of symmetric elements. These long-range neural integration processes have not been explored thoroughly. However, there are some models suggesting two different ways of establishing the required spatial relations in symmetry detection (for review see^6^). Some suggest that perceiving symmetry starts by detecting local information followed by establishing pairs and higher order relations via grouping, while others suggest that it starts from filtered outputs (with filters at multiples spatial scales). In any case, perceiving symmetry requires grouping and integrating symmetric pairs, and our data provides a neural signature for these non-linear integration processes.

The specific non-linear integration processes that we pick up for the PMM wallpaper patterns (with reflection and rotation) and not for the P2 wallpaper patterns (with rotation only) or the feature-matched control stimuli (without reflection and rotation) must be due to the higher-order or larger-scale spatial structures that are unique to reflection symmetry. ‘Grouping’ is a general term that can refer to many different processes (e.g., clustering, segregating, linking, layering, and configuring; see^51^, for further discussion) but the critical grouping that is specific for mirror symmetry must be relatively higher-order than what is available in terms of pairwise links or local clusters in the control conditions.

The size and density of the dots were kept constant in our study. Systematically varying these parameters could potentially modulate the IM amplitudes, and result in a different subset of IMs exceeding the noise floor. Importantly, the size and the density may affect the involvement of different neural networks in a hierarchy covering a different spatial range of integrations. This may, in turn, affect the topography of the symmetry-specific neural activities we reported here. The dependency of the mutual interaction between spatial regions on the size and density of the pattern needs further investigation.

In conclusion, an analysis based on non-linear terms reflecting mutual-interaction between spatial regions revealed differential responses to reflection symmetry that were absent in non-linear terms reflecting non-linear processes associated with each stimulus part. These differential patterns of stimulus selectivity were associated with topographical differences between self and mutual terms that are consistent with a set of sources in early visual areas that are insensitive to symmetry and a distinct set of sources in higher-order visual areas that are sensitive to symmetry.

## Materials and Methods

### Participants

Twenty healthy (12 females, M = 25.15, SD = 9.72) undergraduate students or staff of Stanford University with normal or corrected-to-normal vision participated in the study. Participants were either given course credit or paid for participating. Visual acuity and stereo-acuity were pre-screened and informed consent was provided by participants before the experiment. The Bailey-Lovie chart was used to confirm that participant had normal or corrected-to-normal visual acuity. The RandDot test (http://precision-vision.com/products/stereo-vision-tests/randot-stereo-test.html) was used to confirm normal binocular stereopsis. The Institutional Review Board of Stanford University approved the experimental procedure and the experiment was conducted in accordance with the committee’s guidelines.

### Stimulus presentation

**Figure 6:**
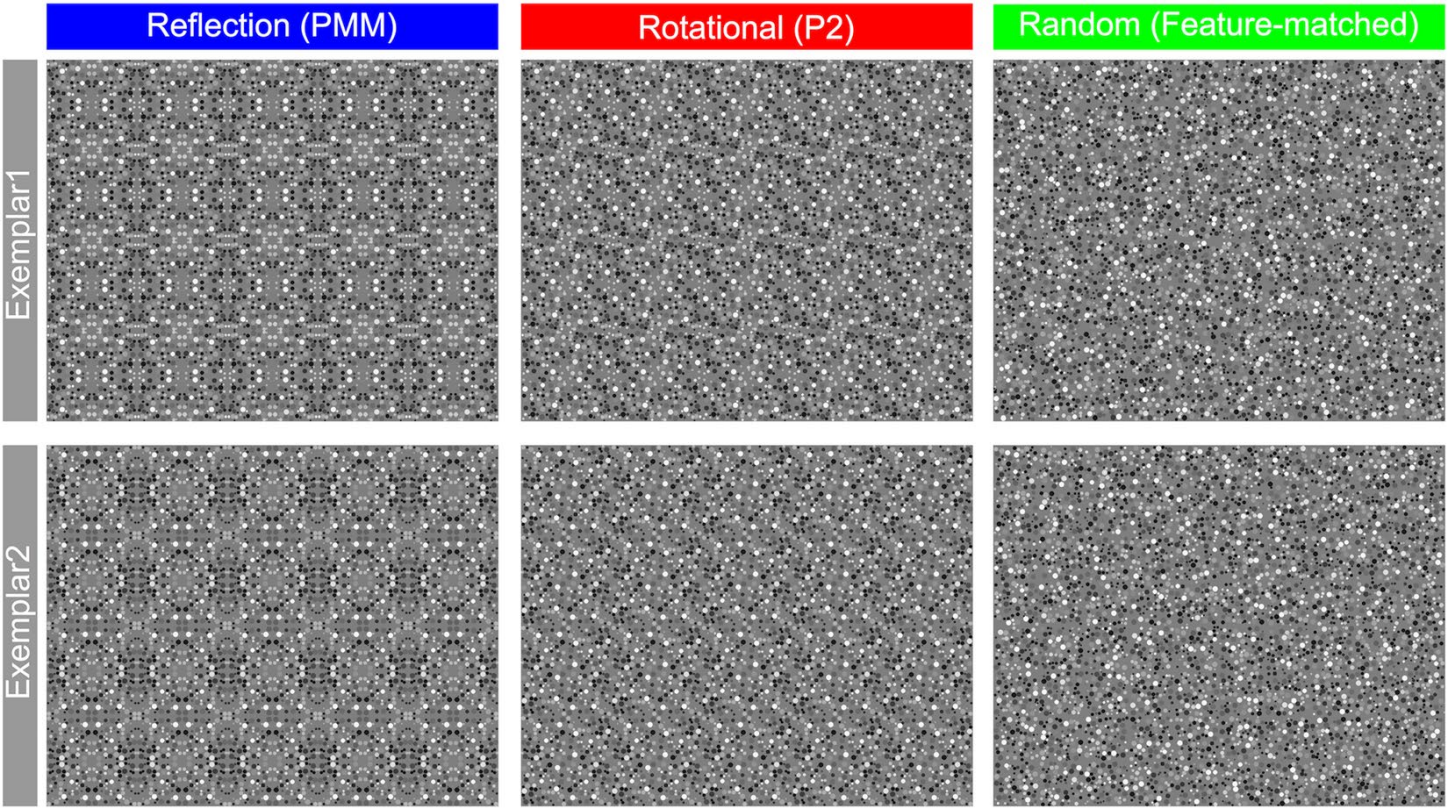
Experiment stimuli. Two exemplars per pattern type were used in the experiment. Rows show two exemplars of three pattern types, while columns show three pattern types used in the experiment. Note that pattern types were renamed for the ease of readers –PMM (Reflection), P2 (Rotational), feature-matched (Random). Also note that the rotational (P2) wallpaper group contains two diagonal rotational symmetry within the unit-pattern, which consists of two 180° rotations but no reflection symmetry.

We generated two exemplars of each of the three pattern types (PMM, P2 and feature-matched) (Fig. 6).

The stimuli were presented using PowerDiva Video software (version 4.8.5, Stanford University) on a 24.5 inch Sony Trimaster EL PVM-2541 display, with a refresh rate of 60 frames per second and resolution of 1024 × 1280 pixels. The size of the individual stimuli was 31° × 38° of visual angle (70 cm viewing distance, contrast 50% and the mean luminance 35 cd/m^2^). The rest of the screen was black.

### EEG frequency-tagging

**Figure 7:**
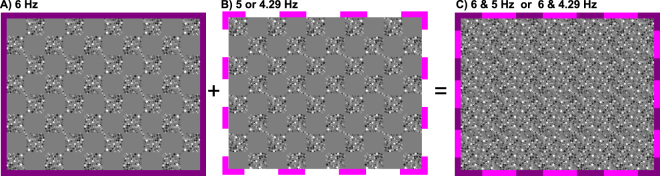
Frequency tagging. An example of one of the image pairs of the P2 condition, which was tagged with two different frequencies. (**A**) The solid purple frame indicates that the first half of the image pair was tagged with 6 Hz. (**B**) The dashed pink frame indicates that the second half of the image pair was tagged with either 5 or 4.29 Hz. Please note that purple/pink lines are only drawn here to explain the symmetry configurations and the frequency tagging method and are not a part of the stimulus presentation. (**C**) By combining these two image pairs, we generated a wallpaper pattern which consists of two frequencies, indicated here as the combination of purple and pink frames.

The contrast of the two diagonally split image-pairs was modulated sinusoidally at two different frequencies (Fig. 7). The frequencies used were integer divisions of the refresh rate of the monitor (f_1_ = 60/10 = 6 Hz and f_2_ = 60/12 = 5 or 60/14 = 4.29 Hz). The two tagging frequencies, f_1_ and f_2_ were each assigned to one of the two image pairs. This diagonal tagging approach eliminated the possibility that EEG signals reflect hemispheric dominance for one of the two input frequencies.

### Experimental procedure

There were six conditions in total (3 pattern types × 2 Freq. Pairs). We ran the trials in random order in three blocks and repeated each condition 10 times. Each trial began with a fixation cross and followed by an exemplar pattern belonging to one of the three types (PMM, P2 or feature-matched) in which each image part was updated at a different frequency. A fixation cross was placed in the middle of the screen and remained on the screen until the end of each trial. To engage attention and to assay perceptual performance, the participants performed a pattern discrimination task on the SSVEP stimuli. For each pattern type, the stimulus presentation started with one of the two exemplars, and then changed at unpredictable times to the other exemplar from the same pattern type, before changing back after 1000 ms or 1166 ms depending on the frequency pair in use. The exemplar change occurred two times, at randomly selected time, within each trial (see Supplementary Movie S1–3). Participants were asked to report the pattern change by pressing a button on a joystick. A printed version of both exemplars from each condition was shown to the participants prior to the experiment to familiarize them with the discrimination task. For each condition, half the trials started with exemplar one, and the other half with exemplar two. Each condition was repeated 30 times in total, so the total number of pattern change was 60 per participant. The trials were initiated by the observer via a button press. Each trial was presented for 10 or 12.8 seconds depending on the frequency pair in use; the inter-trial interval was 2000 ms, minimum.

### EEG acquisition and preprocessing

The EEG data were collected using 128-sensor HydroCell Sensor Nets (Electrical Geodesics) and was bandpass filtered at 0.3 to 50 Hz upon export of the data. The data were analyzed offline, using PowerDiva Host 4.8.5 software. Raw data were subjected to an off-line sample-by-sample thresholding procedure in which noisy and dead sensors were replaced by the average of the six nearest spatial neighbors. For each participant, on average, <10% of the electrodes which were mostly located near the ears or frontal, were replaced. The epoch containing artifacts (ranging between 30 and 50 μV) were eliminated from the further analysis. The entire epoch, number of segments extracted from continuous EEG signal, was eliminated if more than 7 channels had a peak artifact (100 μV). We then re-referenced the EEG data to the common average of all electrodes. The data were segmented into a mutual cycle of both frequencies per frequency pairs (1000 ms for Freq. Pair1; 1166 ms for Freq. Pair2). For instance, 10 second data were segmented into ten 1000 ms epochs for Freq. Pair1. For frequency domain analysis, the complex-value of Fourier coefficients were then averaged across all epochs per repetition, per condition separately for each participant. The EEG data were then transformed into the frequency domain using discrete Fourier transform (DFT). This transformation induced frequency resolution of δf = 0.5 Hz for Freq. Pair1 and δf = 0.43 Hz for Freq. Pair2. For the time domain analysis, we computed the amplitude, cosine and sine values per condition and trial separately for each participant.

### RCA-Frequency Domain

We selected 16 frequencies (6 self-terms (f_1_, f_2_, 2f_1_, 2f_2_, 3f_1_, 3f_2_) and 10 mutual-terms (f_1_ − f_2_, f_1_ + f_2_, f_1_ − 2f_2_, 2f_1_ − f_2_, f_1_ + 2f_2_, 2f_1_ + f_2_, f_1_ + 3f_2_, 3f_1_ + f_2_, 3f_1_ − f_2_, 3f_2_ − f_1_)) as input to the RCA, which was performed in Matlab (MathWorks Inc., Natick, MA) using in-house code. The averages used for plotting were computed as vector averages of real and imaginary values of all participants.

### RCA-Time Domain

Our time domain analysis was based on “IM clean” waveforms, which contained only mutual term information. These waveforms were computed by first filtering the data in the frequency domain so that mutual-terms that were not equal to any of the self-terms up to 50 Hz were included and everything else was excluded. The filtered signals were then inverse-transformed into the time domain and RCA was run on this inverse transformed signals in time domain. After running RCA, we tested for pair-wise waveform differences between pattern types (PMM – P2; PMM – feature-matched; P2 – feature-matched) and ran a permutation testing procedure to correct for multiple comparisons, based on methods devised by Blair and Karniski (see^52^) and described in detail by Appelbaum and colleagues (see^53^).

Briefly, this procedure tested the null hypothesis that no differences were present between pattern types, by generating artificial data sets where the two pattern type labels were permuted randomly across participants. For each of the 1000 permutations the longest run of consecutive time points, where the paired t-test returned p-values < 0.05, was computed to generate a non-parametric reference distribution. The null hypothesis was rejected for any run in which the non-permuted data exceeded 95% of the values in the null distribution. Because each permutation sample contributes only its longest significant sequence to the reference distribution, this procedure implicitly compensates for the problem of multiple comparisons and is a valid test for the omnibus hypothesis of no difference between the waveforms at any time point. Furthermore, this test not only detected significant departures from the null hypothesis, but also localized the time periods when such departures occurred. However, since the correction procedure was tied to the length of the data and the somewhat arbitrary choice of keeping family-wise error at 5%, we also presented the uncorrected significance values (see red/yellow color plots in Fig. 5). By applying both statistical approaches, we were better able to identify time periods when the responses depart from the null hypothesis.

## Electronic supplementary material

PMM

P2

Feature-Matched
